# Handheld Biosensor System Based on a Gradient Grating Period Guided-Mode Resonance Device

**DOI:** 10.3390/bios14010021

**Published:** 2023-12-30

**Authors:** Chien Chieh Chiang, Wen-Chun Tseng, Wen-Tsung Tsai, Cheng-Sheng Huang

**Affiliations:** Department of Mechanical Engineering, National Yang Ming Chiao Tung University, Hsinchu 30010, Taiwan; chieh0128.en10@nycu.edu.tw (C.C.C.); hchstony.en10@nycu.edu.tw (W.-C.T.); tsung.en07@nycu.edu.tw (W.-T.T.)

**Keywords:** handheld biosensor device, optical biosensor, guided-mode resonance, albumin, creatinine

## Abstract

Handheld biosensors have attracted substantial attention for numerous applications, including disease diagnosis, drug dosage monitoring, and environmental sensing. This study presents a novel handheld biosensor based on a gradient grating period guided-mode resonance (GGP-GMR) sensor. Unlike conventional GMR sensors, the proposed sensor’s grating period varies along the device length; hence, the resonant wavelength varies linearly along the device length. If a GGP-GMR sensor is illuminated with a narrow band of light at normal incidence, the light resonates and reflects at a specific period but transmits at other periods; this can be observed as a dark band by using a complementary metal oxide semiconductor (CMOS) underneath the sensor. The concentration of a target analyte can be determined by monitoring the shift of this dark band. We designed and fabricated a handheld device incorporating a light-emitting diode (LED) light source, the necessary optics, an optofluidic chip with an embedded GGP-GMR sensor, and a CMOS. LEDs with different beam angles and bandpass filters with different full width at half maximum values were investigated to optimize the dark band quality and improve the accuracy of the subsequent image analysis. Substrate materials with different refractive indices and waveguide thicknesses were also investigated to maximize the GGP-GMR sensor’s figure of merit. Experiments were performed to validate the proposed handheld biosensor, which achieved a limit of detection (LOD) of 1.09 × 10^−3^ RIU for bulk solution measurement. The sensor’s performance in the multiplexed detection of albumin and creatinine solutions at concentrations of 0–500 μg/mL and 0–10 mg/mL, respectively, was investigated; the corresponding LODs were 0.66 and 0.61 μg/mL.

## 1. Introduction

Commonly used biosensors are based on a fluorescence-based detection mechanism. This mechanism requires labeling with fluorescent dyes, which might hinder the binding of analytes, cause contamination during disposal or cleaning, or degrade over time [1]. By contrast, label-free (LF) biosensors do not have these problems and are highly sensitive and capable of both rapid measurements and real-time monitoring. Therefore, LF biosensors have attracted considerable attention in various fields, including disease diagnosis, environmental monitoring, drug development, drug dosage monitoring, and biowarfare agent and biochemical detection [2,3]. Scholars have fabricated LF biosensors based on various detection mechanisms, including mechanical, electrochemical, acoustic, and optical techniques [4]. Optical LF biosensors are among the most commonly used devices for detecting biomolecules because of their advantages, including their high sensitivity, multiplexing capability, freedom from electromagnetic interference, remote sensing ability, and simple experimental setup [3,4,5,6,7]. Studies have implemented several types of optical LF biosensors, including biosensors based on photonic crystals, ring resonators, waveguides or fibers, interferometry, surface plasma resonance (SPR), localized SPR, guided-mode resonance (GMR), and resonant waveguide grating [2,3,4,5,8]. In particular, SPR-based biosensors are the most widely explored and have been commercialized by numerous companies [9]. However, GMR-based LF biosensors exhibit a higher resolution and a larger figure of merit (FOM) than SPR-based biosensors [1,10] and have a simple readout design; they have thus attracted considerable research attention and have been commercialized by several companies as desktop systems for high-throughput applications [1].

In 2017, Triggs et al. designed a novel chirped GMR biosensor based on a graded duty cycle and demonstrated its performance in detecting the binding between IgG and anti-IgG [11]. Subsequently, the group refined the surface functionalization using polyethylene glycol, achieving an impressive low detection limit of 10 pg/mL [7]. In 2016, our group proposed a linear variable bandstop filter based on a gradient grating period guided-mode resonance (GGP-GMR) [12]. This GGP-GMR filter was subsequently validated as a refractive index (RI) sensor [13] and a potential biosensor [14]. The chirped GMR or GGP-GMR converts spectral information into spatial information such that the signal can be directly read out by a charge-coupled device or complementary metal oxide semiconductor (CMOS) without a bulky spectrometer. In the present study, we leveraged this feature and integrated a GGP-GMR biosensor with a CMOS to develop a first prototype of handheld biosensor. Specifically, we designed and fabricated a handheld device that incorporates the necessary optics and CMOS. For the first time, we investigated the impact of incident light bandwidth and diverging angle on the sensing performance of a GGP-GMR sensor. Additionally, optimization of the GGP-GMR, involving different substrate materials and waveguide thicknesses, was conducted. An optofluidic chip comprising two microfluidic channels with embedded GGP-GMR sensors was also designed; the chip can be inserted into the handheld device for signal readout. To demonstrate the sensor’s multiplexing capability, this study selected albumin and creatinine solutions as test models.

## 2. Materials and Methods

### 2.1. Design of the GGP-GMR Sensor

GMR sensors have undergone thorough exploration since the late 1980s [15,16,17]. Ongoing research efforts persist in investigating novel designs to enhance sensitivity, quality, or FOM [10,18,19,20,21,22]. Additionally, GMR sensors continue to find implementation in emerging biosensing applications [23,24,25,26]. If the device dimensions and optical properties are correctly selected, including grating period, depth, duty cycle, waveguide thickness, and waveguide and cladding material RIs, external illumination at certain incident wavelengths (*λ*) and angles (*θ*) from the cover layer (RI = *n_c_*) can excite the device resonance in accordance with the second-order Bragg condition [27]:

(1) $$n_{c}\sin\theta =\frac{\lambda }{\Lambda }-n_{\mathrm{eff}}$$

where Λ is the grating period, and *n_eff_* is the effective RI of the structure, which is affected by the RIs of the substrate, waveguide layer, and cover (or sample) and by the waveguide thickness [28]. At normal incidence (*θ* = 0), a GMR sensor functions as a bandstop filter that reflects back a specific (or resonant) wavelength of light; light of other wavelengths is transmitted. Hence, if *θ* = 0, Equation (1) can be reduced, and the resonant wavelength can be calculated as follows:

(2) $$\lambda =n_{\mathrm{eff}}\Lambda$$

By varying the grating period along the device length, our group developed a GGP-GMR device that essentially functions as a linear variable bandstop filter; the resonant wavelength also varies along the device length [12]. This device has subsequently been employed in spectral, displacement, fluorescent, and RI measurement applications as well as in LF biosensing [13,14,29,30,31].

When a narrow band of light is incident on a GGP-GMR device, the light resonates at a specific grating period (i.e., at a specific location on the device), as defined in Equation (2); thus, the light is reflected at this period and transmitted at other periods. Light has minimum transmission intensity at this location; this manifests as a dark band when measured with a CMOS device. If the RI (or concentration) of a sample on the GGP-GMR surface changes, *n_eff_* also changes. Therefore, for Equation (2) to be satisfied, the light must resonate at another period, and the dark band shifts accordingly.

### 2.2. Fabrication of an Optofluidic Chip

To demonstrate the performance of the proposed handheld device in multiplexing detection, this study designed and fabricated an optofluidic two-channel microfluidic chip with an embedded GGP-GMR sensor array. Fabricating an optofluidic chip involves three key steps: fabricating a GGP-GMR sensor, fabricating a two-channel microfluidic chip, and bonding these two devices.

Three techniques were used to fabricate the GGP-GMR sensor: electron beam lithography (EBL), replication molding, and film deposition. The design and fabrication processes were similar to those described in our previous work [14]. In brief, a grating pattern with periods from 410 to 450 nm, duty cycle of 0.5 approximately, and depth of 98 nm in 2 nm increments was created using EBL and reactive ion etching on a Si wafer. Each period was repeated 100 times. The GGP pattern was then transferred to an ultraviolet (UV)-curable optical adhesive (Norland 13825) with polyethylene terephthalate (PET) as a substrate. Finally, a layer of TiO_2_ (98 nm) was sputter-deposited to complete the process.

The two-channel microfluidic chip was fabricated using a typical polydimethylsiloxane (PDMS; Sylgard 184, Dow Corning, Midland, MI, USA) replica molding process. First, a poly(methylmethacrylate) (PMMA) mold was manufactured using a micromilling machine. Liquid PDMS with a base-to-curing agent ratio of 8:1 was then poured on top of the PMMA mold. A degassing process was next executed to remove air bubbles, after which the PDMS/PMMA mold was cured at 100 °C for 1 h in an oven. Finally, PDMS was removed from the PMMA mold, and inlet and outlet holes for sample delivery were created with a biopsy punch.

Uncured PDMS was used as an adhesive to irreversibly bind the PDMS microfluidic chip to the GGP-GMR sensor. In brief, a thoroughly mixed and degassed PDMS mixture with a base-to-curing agent ratio of 10:3 was spin-coated on a glass slide at 3000 rpm for 60 s. The microfluidic chip was gently pressed on top of the spin-coated PDMS to pick up a thin a layer of uncured PDMS. The microfluidic chip was then placed on top of the GGP-GMR sensor. The integrated optofluidic chip was completed after curing at 70 °C for 1.5 h in an oven.

### 2.3. Assay Protocol

Albumin and creatinine are commonly used biomarkers for identifying chronic kidney disease and microalbuminuria [32,33]. Accordingly, albumin and creatinine were selected to demonstrate the multiplexing detection performance of the fabricated optofluidic chip with direct readout from the proposed handheld device. First, the GGP-GMR sensor chip was treated with oxygen plasma to generate hydroxyl groups on the TiO_2_ surface, thus enabling epoxy silane (2% 3-glycidoxypropyl triethoxysilane in toluene) to form covalent bonds on the sensor surface. After the surface treatment, the sensor chip was bonded to a microfluidic chip as described in the previous section.

To apply the two-channel microfluidic chip and demonstrate the performance of the proposed handheld device in multiplexing detection, a solution of 100 μg/mL of anti-albumin antibodies (ab10241, Abcam, Cambridge, UK) or anti-creatinine antibodies (ab30719, Abcam) in phosphate-buffered saline (PBS) was injected to cover one of the sensors in each channel, followed by incubation for 12 h at room temperature. Notably, in each channel, the sensor with antibodies was used for measurement, and the sensor without antibodies was used as a reference to correct for any fluctuation. After the incubation process, the antibody solutions were aspirated, and PBS containing 0.05% Tween (PBS-T) was used to rinse the microfluidic channels, removing the unbound antibodies. The channels were then blocked for 1 h by using 1% casein in PBS (Casein Blocker, Bio-Rad, Hercules, CA, USA) to reduce the subsequent nonspecific binding. Subsequently, the blocking solution was extracted from the channels, followed by rinsing with PBS-T. Finally, PBS was injected into the channels and measured. The PBS solution was considered to have 0% concentration, and the results served as the baseline signal.

### 2.4. Detection Principle and Handheld Device

When a narrow band of light is incident on a GGP-GMR sensor, the light is reflected at a specific period, causing the CMOS below to register a dark band. In this study, to fabricate a miniaturized handheld device, a light-emitting diode (LED; KED351 RHD, Kyoto Semiconductor, Kyoto, Japan) was selected as the light source. LEDs typically have a bandwidth on the order of several tens of nanometers, resulting in an excessively broad dark band at the CMOS. To achieve a narrower dark band and thus higher measurement accuracy, a narrow bandpass filter (BF; 656.8-1, Alluxa, Santa Rosa, CA, USA) was incorporated into the handheld device. The grating structure was sensitive to polarization; hence, a polarizer was also required to achieve the desired polarization, improved sensitivity, and narrower dark bands. In addition, we designed a slot to insert the GGP-GMR sensor. A CMOS camera (SME-B050-U, MIGHTEX, Pleasanton, CA, USA) with a built-in readout circuit, a resolution of 2560 × 1920, and a pixel size of 2.2 × 2.2 μm^2^ was used to capture the transmitted images.

**Figure 1:**
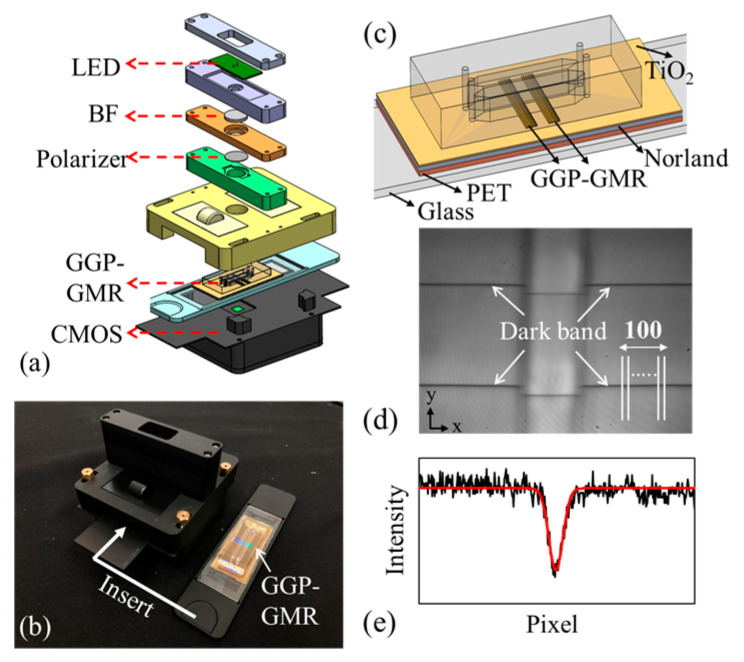
(**a**) Exploded-view diagram of the device. (**b**) Photographs of the handheld device and optofluidic chip. (**c**) Two-channel optofluidic chip with embedded GGP-GMR sensors. (**d**) CMOS image depicting four dark bands within the two channels. (**e**) Raw data (black) and the corresponding fitted intensity distribution (red) along the white line in (**d**).

Figure 1a presents an exploded-view diagram of the fabricated handheld device, including the holders for the components. The device was made from aluminum and manufactured using a computer numerical control machine. These parts were further anodized for enhanced mechanical strength and dyed black to minimize stray light (Figure 1b). Figure 1c presents a schematic of the two-channel optofluidic chip, along with the two GGP-GMR sensors at the bottom of each channel. Moreover, Figure 1d displays an image captured by the CMOS camera, depicting two dark bands in each channel. Figure 1e shows an example of the intensity distribution along one of the white lines in Figure 1d; the smoothdata function in MATLAB was used to fit the raw intensity distribution to a Gaussian model to determine the location of the dark band (minimum intensity in the *y* direction, as illustrated in Figure 1d). In general, the presence of defects or contamination in a GGP-GMR sensor or PDMS microfluidic channels can lead to curve-fitting errors, resulting in an inaccurate determination of the location of a dark band. To alleviate this problem, we used an averaging technique; specifically, we derived the average location of the dark band along the *y* direction from 100 lines (Figure 1d) to represent the location of the dark band for each image.

## 3. Results

### 3.1. Bulk Solution Measurement

As mentioned, if the RI of a sensor surface varies, *n_eff_* of the overall structure changes accordingly. Consequently, the incident wavelength resonates at a different period, in accordance with Equation (2), causing the dark band on the CMOS to shift. To characterize the sensor performance in terms of bulk sensitivity and limit of detection (LOD), this study used sucrose solutions of various concentrations. First, deionized (DI) water was injected into both channels, and images were captured every 10 s for 5 min. A sucrose solution was then injected into one of the channels (measurement channel) at a concentration from 10% to 60% in 10% increments. Before sucrose at another concentration was injected into the measurement channel, the previously injected solution was aspirated, and the channel was rinsed with DI water. The other channel was used as a reference; only DI was injected into the reference channel. The results obtained for the measurement and reference channels are indicated as MEA and REF, respectively, in the subsequent figures presented herein.

**Figure 2:**
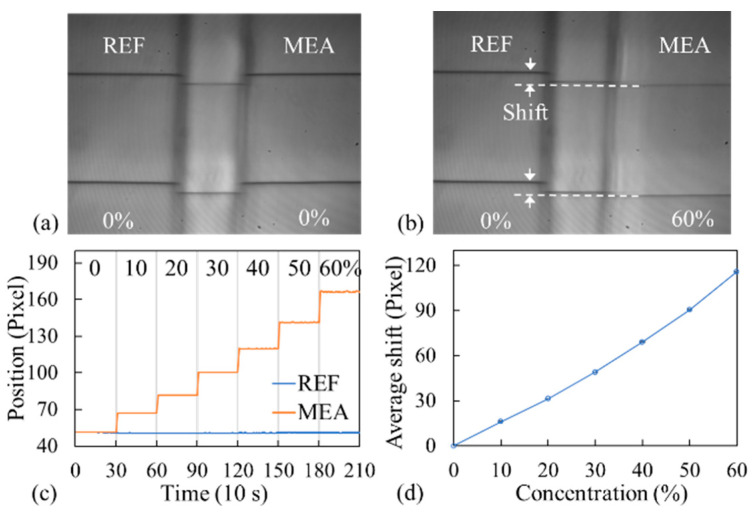
CMOS images for (**a**) 0% and (**b**) 60% sucrose solutions in the measurement channel. (**c**) Locations of the dark bands at various sucrose concentrations measured by one sensor in each of the measurement and reference channels. (**d**) Net average shift in the dark bands as a function of sucrose concentration.

Figure 2a depicts a CMOS image that was captured when both channels were filled with DI water (0%); two dark bands were visible within each channel. Figure 2b presents an image that was captured when the measurement channel was filled with 60% sucrose; the dark band observed in the measurement channel exhibited a shift relative to that in the reference channel. Figure 2c displays the locations of the dark bands observed in the two channels at various sucrose concentrations for each of the top two sensors in Figure 2a. For the measurement channel, the net shift of the bands at each concentration relative to the measurement at a sucrose concentration of 0% was calculated by subtracting the shift of the reference channel band from the shift of the measurement channel band; hence, two sets of data (top and bottom sensors in Figure 2d) were obtained from a single optofluidic chip. The entire processes were repeated using another optofluidic chip, and the average net shift, along with the standard deviation as a function of sucrose concentration, was derived, as presented in Figure 2d.

The average total net shift observed at a sucrose concentration of 60% relative to the measurement at a concentration of 0% was 115.86 pixels (254.89 μm). The change in RI for sucrose concentrations from 0% to 60% was 0.1088 [34]. The average sensitivity (0–60%), calculated as the amount of shift divided by the amount of change in RI (RIU), was 2342.75 μm/RIU. The average of the standard deviation of all the concentration measurements from the four sets of data was 0.85 μm. The LOD, calculated as three times the standard deviation divided by the sensitivity, was 1.09 × 10^−3^ RIU.

### 3.2. Optimization of the Optical Setup and GGP-GMR Sensor

**Figure 3:**
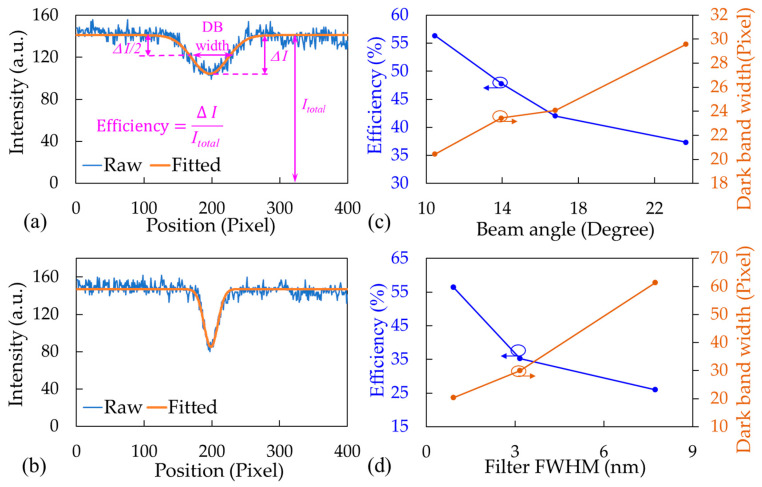
Dark band intensity distributions for (**a**) LED with a BA of 10.45° and a BF with a FWHM of 7.74 nm and (**b**) LED with a BA of 16.78° and a BF with a FWHM of 0.94 nm. (**c**) Relationship between dark band width and resonant efficiency for (**c**) the same BF and LEDs with different BAs and (**d**) BFs with different FWHM values and the same LED.

The bulk sensitivity of a GGP-GMR sensor represents the magnitude of the sensor response to a given change in sample concentration, as described in the previous section. However, dark band narrowness is also a critical parameter for sensor resolution, which is the minimum resolvable difference between two measurements. Narrower dark bands are desirable because measuring small shifts is easier. The narrowness of a dark band is analogous to the resonant bandwidth in many optical resonator biosensors. Hence, the figure of merit (FOM), which is used to evaluate typical optical resonator biosensors [10,35], can also be used to evaluate a GGP-GMR sensor. For the GGP-GMR sensor in this study, the FOM was defined as the ratio of sensitivity to the dark band width. A higher FOM indicates superior sensing performance in terms of both sensitivity and resolution. In addition, the resonant efficiency was defined as the reduced intensity at the dark band divided by the intensity at a nonresonant location, as shown in Figure 3a. At a higher resonant efficiency, more light is reflected at a resonant period, resulting in a lower intensity at the dark band. Essentially, the resonant efficiency indicates the darkness of the dark band. A darker dark band reduces the effects of signal noise, defects, or contamination from the GGP-GMR or PDMS device, improving both the accuracy of the curve fitting process and the accuracy of the dark band location derived through image analysis.

The width of a dark band can be affected by both the GGP-GMR sensor itself and the detection setup. In this study, we investigated how the beam angle (BA, defined as the diverging angle at 50% of maximum intensity) of the LED and the full width at half maximum (FWHM) of the BF affect the dark band’s width and resonant efficiency. Moreover, sensitivity can be considered an intrinsic property of the GGP-GMR sensor itself. For a conventional GMR sensor (fixed grating period), a substrate with a lower RI can shift the waveguide mode toward the cladding layer (i.e., the sample solution); this can increase the portion of the evanescent field in the cladding layer that interacts with the analyte, thereby increasing the sensor’s sensitivity [36,37]. However, this would also broaden the resonant bandwidth, reducing performance in terms of the FOM. In this study, we investigated how various substrate materials (i.e., materials with different RIs) affect the sensitivity and dark band quality of a sensor. We also investigated the effects of TiO_2_ thickness on the broadening of the dark band’s width to maximize the FOM.

#### 3.2.1. Effects of LED BA and BF Bandwidth

Figure 3a presents two examples of the intensity distributions of dark bands from two sets of LEDs and BFs. A BF with an FWHM of 7.74 nm and an LED with a BA of 10.45° resulted in dark band width and efficiency of 61.38 pixels and 26.01, respectively (Figure 3a). By contrast, a BF with an FWHM of 0.94 nm and LED with a BA of 16.78° resulted in dark band width and efficiency of 24.09 pixels and 42.02, respectively (Figure 3b).

The preceding results indicated that the LED BA and BF FWHM considerably influenced the widths and efficiency levels of the dark bands. To further investigate these effects, two sets of experiments were conducted. First, we investigated the effect of the LED BA on dark band quality when a fixed BF FWHM of 0.94 nm was used. We tested four commercial LEDs with a center wavelength of 660 nm, namely, CMD333 URC-2 (Visual Communications, Carlsbad, CA, USA), MTE5066CJ-UR (Marktech Optoelectronics, Latham, NY, USA), MTE5066S1J-UR (Marktech Optoelectronics), and KED351RHD (Kyoto Semiconductor, Kyoto, Japan); these LEDs had measured BAs of 23.65°, 16.78°, 13.94°, and 10.45°, respectively. Figure 3c presents a summary of the test results, indicating that LEDs with smaller BAs resulted in narrower dark bands and higher resonant efficiency levels.

We next investigated the effect of the BF FWHM on dark band quality when the best-performing LED (KED351RHD with a BA of 10.45°) was used. Three commercial BFs were tested, namely, Edmund Optics 656-10 (measured center wavelength and FWHM of 657.37 and 7.74 nm, respectively), OptoSigma 671-3.65 (measured center wavelength and FWHM of 672.23 and 3.17 nm, respectively), and Alluxa 656.8-1 (measured center wavelength and FWHM of 656.1 and 0.94 nm, respectively). Figure 3d illustrates the test results, indicating that narrower FWHMs resulted in narrower dark bands and higher resonant efficiency levels. Therefore, the LED with the smallest BA (KED351RHD) and the BF with the smallest FWHM (Alluxa) were selected for integration into the portable device for the subsequent sucrose and biomolecule measurements.

#### 3.2.2. Substrate RI and Waveguide Thickness

Two Norland optical adhesives, namely, NOA 13825 (RI = 1.3825) and NOA68 (RI = 1.54), were selected to investigate how the RI of a substrate affects the sensitivity of a GGP-GMR sensor. In addition, the effects of five TiO_2_ thicknesses (from 50 to 130 nm in increments of 20 nm) on the quality of the dark bands were investigated. The combination of substrate and TiO_2_ thickness that resulted in the largest FOM was selected to determine the LOD for the bulk solution measurement and the multiplexing detection of biomolecules.

**Figure 4:**
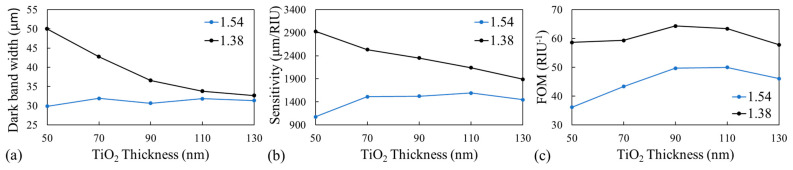
(**a**) Dark band width, (**b**) sensitivity, and (**c**) FOM as a function of TiO_2_ thickness for substrates with RIs of 1.38 and 1.54.

DI water was injected into different GGP-GMR sensors (i.e., with different substrates and TiO_2_ thicknesses), and the widths of the corresponding dark bands were measured. Figure 4a displays a summary of the measurement results. The results revealed that for the substrate with an RI of 1.38, the dark band width decreased as the TiO_2_ thickness increased. By contrast, for the substrate with higher RIs, the dark band width was narrower overall but was not greatly affected by the TiO_2_ thickness. Therefore, a narrower band width is best achieved by selecting a substrate with a higher RI.

To further investigate the effects of substrate RI and TiO_2_ thickness on the sensitivity of the GGP-GMR sensor, sucrose solutions of varying concentrations (from 0% to 60% in increments of 10%) were used. The process was the same as that described for the bulk solution measurement in Section 3.1. Figure 4a presents a summary of the measurement results. Overall, the GGP-GMR sensor had higher sensitivity when the substrate RI was lower; this result is consistent with the behavior of conventional GMRs [38]. When the RI was 1.38, the sensitivity increased as the TiO_2_ thickness decreased. To optimize both sensitivity and detection resolution, the FOM was used to select the combination with the best sensing performance. The GGP-GMR with the NOA13825 substrate and TiO_2_ thickness of 90 nm had the highest FOM (Figure 4c) and was thus selected to characterize the LOD for bulk solution measurement and biomolecule detection.

### 3.3. Biomolecule Detection

As mentioned, albumin and creatinine are commonly used biomarkers for microalbuminuria detection. These molecules were thus selected for tests to demonstrate the multiplexing capability of the proposed handheld biosensor device. As displayed in Figure 1c,d, one channel was used to measure albumin, and the other was used to measure creatinine. Within each channel, one of the sensors was immobilized with antibodies and used as a measurement sensor, and the other (without antibodies) was used as a reference sensor. Four dark bands corresponding to each of the four sensors could be captured simultaneously; hence, both albumin and creatinine could be measured concurrently. Standard curves were established for various concentrations of albumin (ab205808, Abcam) and creatinine (ab143309, Abcam) in 1% casein in PBS. Albumin solutions of five concentrations (from 500 to 0.8 μg/mL) with fivefold dilution were used; a blank solution (0%) was also used. Creatinine solutions of concentrations ranging from 10 mg/mL to 1 μg/mL with 10-fold dilution were used; a blank solution was also used.

**Figure 5:**
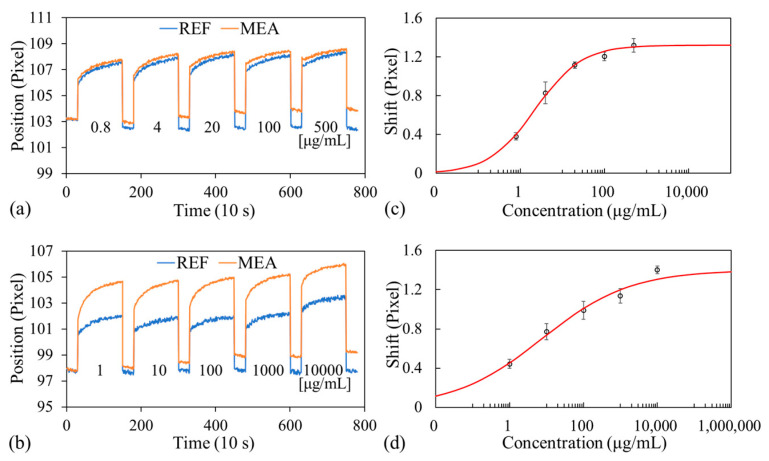
Dark band locations as a function of time (or analyte concentration) for both reference and measurement sensors for the (**a**) albumin and (**b**) creatinine channels. Dose–response curves for (**c**) albumin and (**d**) creatinine.

Figure 5a presents the locations of the dark bands with time (or concentration) for both the reference and the measurement sensors observed for the albumin channel in one experimental run. Initially, PBS (blank) was injected into both channels, and images were captured every 10 s for 5 min; the obtained locations of the dark bands were considered the baseline signals. Then, PBS was aspirated, and the albumin and creatinine solutions (starting from the lowest concentration) were injected into their respective channels, covering both sensors in each channel. Images were captured every 10 s for 20 min. The solutions were aspirated, and the channels were rinsed with PBS-T to remove unbound or loosely bound antigens. Finally, fresh PBS was injected, and the transmitted images were captured every 10 s for 5 min (valley regions in Figure 5a,b). Signals obtained at this step indicated that the dark bands shifted relative to the baseline signals (blank signal) owing to the binding between the target analytes and the immobilized antibodies; these signals were used for our subsequent analysis. This procedure was repeated for each concentration.

The net shift of the dark band caused by the analyte binding with the immobilized antibodies was determined by subtracting the shift of the dark band of the reference sensor (no antibodies) from the shift of the dark band of the measurement sensor (with antibodies) to account for any nonspecific binding and fluctuations caused by the environment, the experimental procedure, or the measurement setup. The standard curves obtained from the three experimental runs using three optofluidic chips are presented in Figure 5c,d. A four-parameter logistic model was used to fit these experimental data by employing OriginPro 2016; the fitted curves (red lines) are also shown in Figure 5c,d. The linear ranges obtained for albumin and creatinine were 0.8–20 μg/mL and 1.0–1000 μg/mL, approximately. The LOD for albumin or creatinine detection using the proposed handheld device was defined as the concentration corresponding to the shift associated with the blank (baseline) concentration plus three times the average standard deviation of the shift associated with all of the measured concentrations from the three experimental runs. The LODs obtained for albumin and creatinine were 0.66 and 0.61 μg/mL, respectively.

### 3.4. Specificity Demonstration

**Figure 6:**
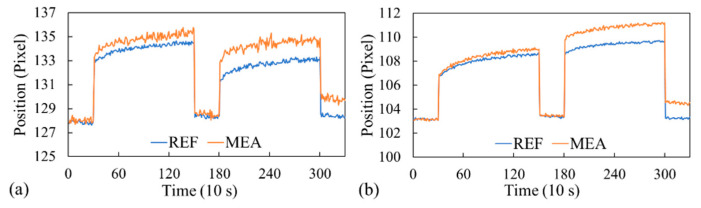
Dark band location versus time for the (**a**) albumin and (**b**) creatinine channels.

We further tested the nonspecific binding of the fabricated optofluidic chip. The optofluidic chip was first prepared as described in Section 3.3. One channel was prepared for albumin, and the other was prepared for creatinine. Within each channel, the measurement sensor was immobilized with antibodies, while the reference sensor did not contain antibodies. We first injected PBS to establish the baseline signal. Images were captured every 10 s for 5 min. The baseline signal (or blank signal) was then calculated as the average location of the dark band from 30 images. Subsequently, 10 mg/mL of creatinine was injected into the albumin channel, followed by incubation for 20 min. Then, creatinine was aspirated, and PBS-T was used to rinse the channel. Finally, PBS was injected, and the average location of the dark bands from 30 images represented the dark band’s location at this step. The shift of the dark band relative to the baseline signal (blank signal) was attributed to the binding between the target analytes and the immobilized antibodies. The net dark band shift was calculated by subtracting the shift of the band of the reference sensor from the shift of the band of the measurement sensor; it was only 0.007 pixel. This implies that the albumin antibodies did not bind to creatinine. The injected PBS was then aspirated, and albumin (500 μg/mL) was injected into the albumin channel, followed by incubation for 20 min. The albumin solution was aspirated, and after the channel was rinsed with PBS-T, PBS was injected for dark band measurement. The dark band observed for the measurement sensor shifted substantially (1.259 pixels) with respect to that observed for the reference sensor, owing to the antibody binding to albumin (Figure 6a).

A similar experiment was performed for the creatinine channel. PBS was first injected to obtain the baseline signals for both the measurement and the reference sensors. Albumin (500 μg/mL) was then injected. After aspiration and washing, PBS was again injected. Similarly, a minuscule net shift (0.082 pixel) of the dark band in the measurement sensor relative to that of the reference sensor was observed (Figure 6b), indicating no or weak binding between the anti-creatinine antibodies and albumin. The albumin solution was then aspirated, the channel was rinsed, and a 10 mg/mL solution of creatinine was injected, followed by incubation for 20 min. The injected creatinine was then aspirated, and the channel was rinsed. Finally, PBS was injected for signal measurement. We observed a clear shift (1.303 pixels) of the dark band of the measurement sensor relative to that of the reference sensor (Figure 6b), indicating binding between the anti-creatinine antibodies and creatinine. A more thorough investigation could be carried out with similar biomolecules (i.e., molecules with a similar molecular weight) to assess the specificity.

## 4. Conclusions

This study designed and fabricated a handheld biosensor with an optofluidic chip comprising two microfluidic channels with an embedded GGP-GMR sensor array. The handheld device was fabricated using aluminum through a computer numerical control machine and was determined to be palm-sized, with a weight of 356 g (approximately twice the weight of a smartphone). The optofluidic chip was fabricated by bonding an inexpensive UV-replicated GGP-GMR sensor chip to a PDMS-replicated microfluidic channel. We used a light source with a small BA and a BF with a small FWHM to narrow the dark band width and increase the resonant efficiency of the sensor, thereby improving the sensor’s detection resolution. We observed that substrates with lower RIs resulted in higher sensitivity levels but were associated with broader dark bands. We also optimized the TiO_2_ thickness, observing that a substrate with an RI of 1.38 and a TiO_2_ thickness of 90 nm could achieve the highest FOM (best sensing performance); these parameters were thus used for the system fabrication. Sucrose solutions were used to characterize the sensitivity and resolution of the optofluidic chip and the readout from the handheld device; an LOD of 1.09 × 10^−3^ RIU was achieved. Furthermore, the optofluidic chip and the portable system were demonstrated to achieve the simultaneous detection of albumin and creatinine, with the LODs for albumin and creatinine in PBS solution being 0.66 and 0.61 μg/mL, respectively. Hence, the implemented sensor could meet the requirements for clinical applications.

The system’s detection capability can be further enhanced in two ways. The results depicted in Figure 3 suggest that using a light source with a narrower bandwidth and a smaller beam angle can reduce the dark band width and enhance the resonant efficiency, improving the LOD. A simple epoxy silane surface functionalization was employed for antibody immobilization to demonstrate biomolecule detection. Further exploration of surface functionalization can be undertaken to enhance the antibody binding density or orientation, consequently improving the antigen binding efficiency and, thereby, achieving a better overall detection sensitivity.

In this study, image acquisition and processing, data analysis, and result visualization for the proposed device were performed on an external laptop. In the future, a custom-made circuit board with a readout circuit, a central processor, and a monitor could be further integrated with the handheld device to build a completely standalone device, which will be useful in many biosensing applications.

## Data Availability

Data available on reasonable request from the corresponding author.
